# Estudo Controlado das Alterações Hemodinâmicas Centrais de uma Sessão de Exercício Inspiratório com Diferentes Cargas na Insuficiência Cardíaca

**DOI:** 10.36660/abc.20180375

**Published:** 2020-05-12

**Authors:** Luana de Decco Marchese, Sergio Chermont, Danielle Warol, Lucia Brandão de Oliveira, Sabrina Bernardez Pereira, Mônica Quintão, Evandro Tinoco Mesquita

**Affiliations:** 1 Universidade Federal Fluminense Niterói RJ Brasil Universidade Federal Fluminense, Niterói, RJ – Brasil; 2 Centro Universitário Serra dos Órgãos Clínica de Insuficiência Cardíaca Teresópolis RJ Brasil Centro Universitário Serra dos Órgãos – Clínica de Insuficiência Cardíaca (CLIC), Teresópolis, RJ – Brasil; 3 Hospital Santa Martha Niterói RJ Brasil Hospital Santa Martha, Niterói, RJ – Brasil; 4 Hospital do Coração São Paulo SP Brasil Hospital do Coração, São Paulo, SP – Brasil; 5 Instituto Nacional do Câncer Rio de Janeiro RJ Brasil Instituto Nacional do Câncer,Rio de Janeiro, RJ – Brasil

**Keywords:** Insuficiência Cardíaca, Debilidade Muscular, Exercícios Respiratórios, Hemodinâmica, Síndrome de Fadiga Crônica, Terapia por Exercício, Qualidade de Vida, Técnicas de Exercício e Movimento

## Abstract

**Fundamento:**

A fraqueza muscular inspiratória contribui para a intolerância ao exercício e diminuição da qualidade de vida dos pacientes com insuficiência cardíaca. Estudos com treinamento da musculatura inspiratória demonstram melhora da força muscular inspiratória, da capacidade funcional e da qualidade de vida. Porém, pouco se sabe sobre a resposta hemodinâmica central (RHC) durante o exercício inspiratório (EI).

**Objetivo:**

Avaliar a RHC em uma única sessão de EI com diferentes cargas (placebo, 30 e 60%) na insuficiência cardíaca.

**Métodos:**

Ensaio clínico randomizado placebo-controlado, em pacientes com insuficiência cardíaca com fração de ejeção reduzida, classe funcional II e III. Vinte pacientes, com idade de 65±11 anos, completaram uma sessão única de exercício inspiratório, em 3 ciclos de 15 minutos, com *washout* de 1 hora, envolvendo cargas de 30% (C30), 60% (C60) e placebo, utilizando um resistor de carga linear ( *PowerBreathe Light* ). O estudo hemodinâmico não invasivo foi realizado por bioimpedância cardiotorácica ( *Niccomo™CardioScreen®* ). Análise estatística foi feita com o Teste *t* *de* Student e a correlação de Pearson, considerado significante p≤0,05.

**Resultados:**

Foi observado aumento da frequência cardíaca (FC) com a C30 (64±15 vs 69±15 bpm; p=0,005) e C60 (67±14 vs 73±14 bpm, p=0,002). No volume sistólico (VS), observou-se diminuição com a C30 (73±26 vs 64±20 ml; p=0,004). O débito cardíaco (DC) apresentou aumento apenas com a C60 (4,6±1,5 vs 5,3±1,7 l/min; p=-0,001).

**Conclusão:**

Quando utilizada a carga de 60%, em uma sessão única de EI, foram observadas alterações na RHC. A FC e o DC aumentaram, assim como as escalas de Borg e sensação subjetiva de dispneia. Já a carga de 30% promoveu diminuição do VS. (Arq Bras Cardiol. 2020; 114(4):656-663)

## Introdução

A maioria dos pacientes com insuficiência cardíaca (IC) apresenta intolerância ao exercício, devido principalmente a sintomas como dispneia e fadiga. Essa baixa tolerância aos esforços gera um ciclo de inatividade física e consequente diminuição da qualidade de vida.^1^

Além de outros mecanismos já descritos, como a excessiva necessidade ventilatória, o ergorreflexo muscular exacerbado e o aumento da atividade simpática, a fraqueza muscular inspiratória, presente em cerca de 30 a 50% dos pacientes com insuficiência cardíaca com fração de ejeção reduzida (ICFER), tem sido apontada como um fator que pode contribuir para a intolerância ao exercício^2 , 3^ e que apresenta valor prognóstico independente.^4 , 5^

Estudos previamente publicados demonstraram que o treinamento da musculatura inspiratória (TMI) resulta em melhorias significativas da força muscular inspiratória, da capacidade funcional, da dispneia e da resposta ventilatória durante o exercício, além de contribuir para melhora da qualidade de vida dos pacientes com IC.^6 , 7^ Porém, a intensidade ideal de treinamento para otimizar esses resultados ainda não está clara. Uma recente revisão sistemática com meta-análise sugere que o TMI de alta intensidade seja superior às menores cargas e parece não apresentar efeitos adversos.^8^

Os estudos concentram-se em demonstrar os benefícios sistêmicos do TMI, porém pouco se sabe sobre a resposta hemodinâmica central (RHC) desses pacientes durante o exercício inspiratório (EI).^9^ A hipótese do presente estudo é que, com uma carga mais alta, seriam observadas maiores repercussões hemodinâmicas. Sendo assim, este estudo teve como objetivo avaliar a RHC em uma única sessão de exercício inspiratório com diferentes cargas (placebo, 30 e 60%) na ICFER.

## Métodos

Ensaio clínico randomizado, placebo-controlado. A carga era colocada no resistor de carga linear, de forma que os participantes não visualizavam em qual nível estava posicionado o marcador e também não eram informados sobre a carga empregada.

### Critérios de inclusão e exclusão

Para atender o objetivo deste estudo, foram selecionados 29 pacientes com ICFER da Clínica de Insuficiência Cardíaca (CLIC) do Centro Universitário Serra dos Órgãos (UNIFESO), que preencheram os seguintes critérios de inclusão: diagnóstico clínico de insuficiência cardíaca, idade acima de 21 anos, ecodopplercardiograma com fração de ejeção do ventrículo esquerdo (FEVE) <45% (método de Simpson), classe II e III pela New York Heart Association (NYHA), enfermidade estável há pelo menos três meses, nunca ter realizado ou não estar em tratamento com TMI. E nenhum dos critérios de exclusão descritos a seguir: diagnóstico clínico (médico) de doença pulmonar obstrutiva crônica, angina instável, arritmias cardíacas importantes, infarto agudo do miocárdio dentro dos últimos três meses, incapacidade de realizar a sessão de EI. E ainda nenhum dos critérios de exclusão da bioimpedância cardiotorácica: derrame pleural volumoso, anasarca, insuficiência aórtica moderada ou grave, uso de balão intra-aórtico, pressão arterial média >130mmHg, altura <1,20m ou >2,30m, peso <30kg ou >155kg, e uso de marca-passos com sensores para ajuste de frequência cardíaca de acordo com a frequência respiratória.

### Métodos de avaliação

Como instrumentos de coleta, foram utilizados: um manovacuômetro analógico ( *Critical Med®* , Brasil), um resistor de carga linear ( *PowerBreathe Light®* , Estados Unidos) e um aparelho de bioimpedância cardiotorácica (BCT) ( *Niccomo™ CardioScreen®* , Alemanha).

As sessões de EMI foram realizadas de acordo com a randomização feita pelo site *Randomizer* , utilizando o resistor de carga linear durante 15 minutos com as seguintes resistências: 0 (placebo), 30% e 60% do valor da pressão inspiratória máxima (PImáx) medida previamente através da manovacuometria, com *washout* de 1 hora. Para acompanhar a repercussão hemodinâmica, foi utilizado o aparelho de BCT.

### Exercício inspiratório

Por ser a primeira vez que os participantes do estudo utilizaram o resistor de carga linear, após a avaliação inicial eles foram orientados sobre como deveriam realizar o EI e, depois, permaneceram 15 minutos em repouso antes de começar a monitorização hemodinâmica.

Seguindo a randomização das cargas feita previamente (placebo, 30% ou 60%), o EI foi realizado durante 15 minutos, com o paciente em posição supina sobre poltrona reclinável, a 45º de elevação. Todos os participantes utilizaram o mesmo resistor de carga linear, porém foi utilizado um filtro individual adquirido com o próprio fabricante, que foi descartado após o experimento.

Ao longo do EI, o indivíduo foi instruído a realizar inspiração e expiração de acordo com o sinal sonoro emitido por um software ( *Paced Breathing* ), de forma que todos os participantes realizaram 15 incursões respiratórias por minuto.^8^ O treinamento com as demais cargas foi realizado após uma hora de intervalo entre cada sessão. Para a realização do EI com placebo, foi retirada a mola do dispositivo, permanecendo somente a válvula unidirecional, não havendo resistência à inspiração do paciente.

### Análise estatística

O número adequado de participantes a serem estudados foi calculado com base em publicações prévias que mostravam qual intervenção, como efeitos do exercício, provocam alterações significativas, tal como aumento da frequência cardíaca, entre outros. Para esta magnitude de efeitos e para fixar o poder estatístico em 0,8 e erro alfa em 0,05, a amostra deveria compreender 20 indivíduos.

Todos os dados foram submetidos à análise de Kolmogorov-Smirnov para determinar se havia ou não distribuição normal da amostra e dos dados. As variáveis hemodinâmicas durante o EI, nos grupos Placebo, EI 30% ou EI 60% foram comparadas pelo teste *t de* Student pareado. Para associação das variáveis independentes, foi utilizada a correlação de Pearson. Quando os valores de p foram significantes, comparações pareadas foram feitas por meio do teste de Bonferroni (post-hoc).

Os dados foram transportados para uma planilha sistemática do programa *Prism GraphPad 5.0 (GraphPad Software, San Diego, CA* *).* As variáveis categóricas foram expressas em números absolutos. Todos os resultados foram expressos em média±desvio padrão e valores de p<0,05 foram considerados estatisticamente significativos.

### Considerações éticas

Todos os participantes deste estudo receberam informações detalhadas sobre a finalidade da pesquisa e os procedimentos a serem realizados. O protocolo foi enviado para o Comitê de Ética em Pesquisa do UNIFESO e aprovado sob o parecer número 420.737, com registro na Plataforma Brasil.

Os pacientes, antes de participarem do estudo, assinaram o termo de consentimento livre e esclarecido, conforme a resolução 466/2012 do Conselho Nacional de Saúde.

## Resultados

**Figura 1:**
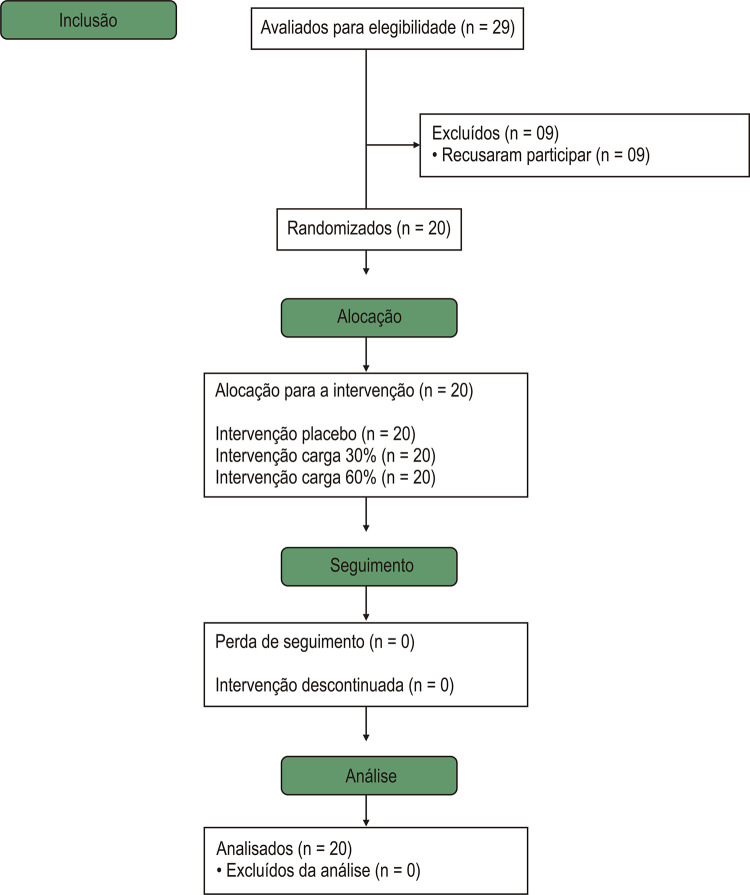
– *Diagrama de fluxo CONSORT.*

Dentre os 29 participantes selecionados para o estudo, 20 concluíram o experimento (nove pacientes recusaram-se a participar) ( Figura 1 ). Na Tabela 1 estão descritas as características demográficas, clínicas e de tratamento farmacológico da amostra estudada.

**Tabela 1:** – Características da amostra

	n *=* 20
Sexo	13H\7M
Idade (anos)	65 ± 11
Peso (kg)	72 ± 14
Altura (cm)	164 ± 11
IMC (kg/m^2^)	26,7 ± 4,4
Etnia	Brancos (7), Pardos (6), Afrodescendentes (7)
NYHA	Classe II (14), Classe III (6)
FEVE (%)	37,2 ± 6,3
PIMáx (cm\H_2_O)	- 101 ± - 43
PEMáx (cm\H_2_O)	95 ± 42
Carga TMI 30% (cm\H_2_O)	31 ± 11
Carga TMI 60% (cm\H_2_O)	61 ± 25
**Terapia farmacológica**	
Inibidor da ECA, %	55
Diuréticos, %	75
β-bloqueador, %	80

*H: homens; M: mulheres; kg: quilograma; cm: centímetros; IMC: índice de massa corpórea; kg/m^2^: quilogramas por metros quadrados; FEVE: fração de ejeção do ventrículo esquerdo; PIMáx: pressão inspiratória máxima; PEMáx: pressão expiratória máxima; TMI: treinamento muscular inspiratório; cm/H_2_O: centímetros de água; ECA: enzima conversora de angiotensina.*

### Respostas das variáveis hemodinâmicas centrais ao EI

**Figura 2:**
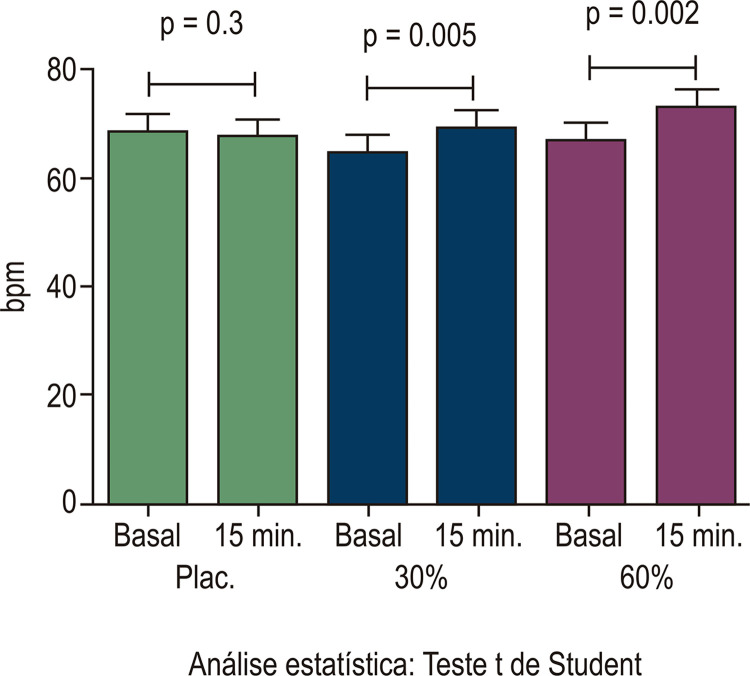
Comportamento da FC pré e aos 15 min. de EI com as diferentes cargas. FC: frequência cardíaca; EI: exercício inspiratório; bpm: batimentos por minuto; plac: placebo; min.: minutos. (Fonte: O autor)

**Figura 3:**
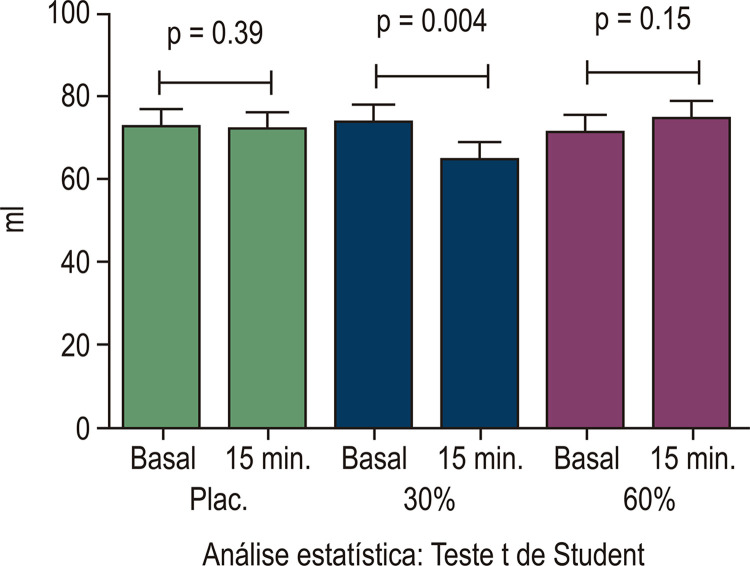
– *Comportamento do VS pré e aos 15 min. de EI com as diferentes cargas. VS: volume sistólico; EI: exercício inspiratório; ml: mililitro; plac: placebo; min.:minutos. (Fonte: O autor)*

**Figura 4:**
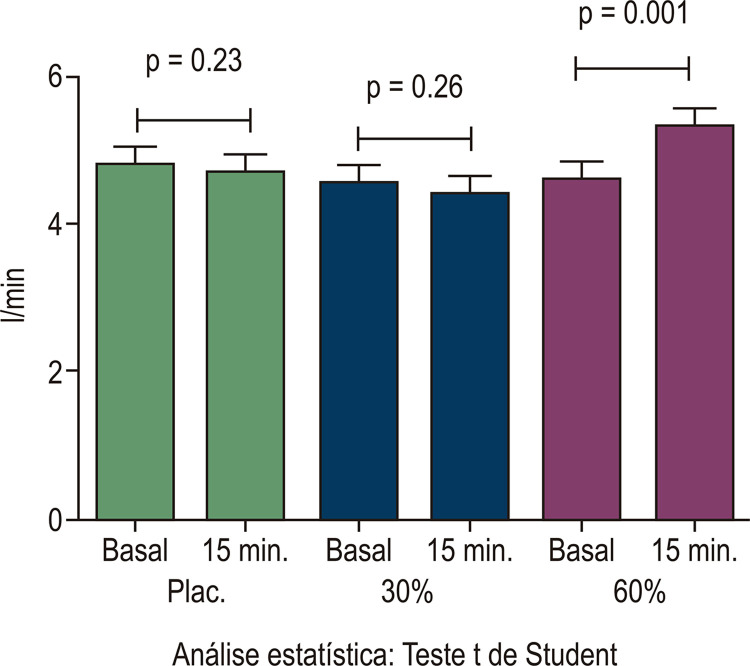
– *Comportamento do DC pré e aos 15 min. de EI com as diferentes cargas. DC: débito cardíaco; EI: exercício inspiratório; ml: mililitro; plac: placebo; min.: minutos. (Fonte: O autor)*

A resposta hemodinâmica central apresentou um comportamento diferente entre as diferentes cargas de EI na amostra estudada. A FC aumentou com as cargas de 30% (C30) (64±15 vs 69±15 bpm; p=0,005) e 60% (C60) (67±14 vs 73±14 bpm, p=0,002), mas não apresentou mudanças no modo placebo (P) ( Figura 2 ). Ocorreu diminuição do VS quando realizado o EI com a C30 (73±26 vs 64±20 ml; p=0,004) e não houve mudanças com as cargas P e C60 ( Figura 3 ). O DC aumentou quando realizado o EI com a C60 (4,6±1,5 vs 5,3±1,7 l/min; p=-0,001) e não apresentou mudanças com as cargas P e C30 ( Figura 4 ).

### Respostas das demais variáveis hemodinâmicas ao EI

Além da RHC, outras variáveis hemodinâmicas também sofreram modificações ao longo do EI com a C60. O placebo e a C30 não geraram nenhuma alteração nas variáveis apresentadas.

**Figura 5:**
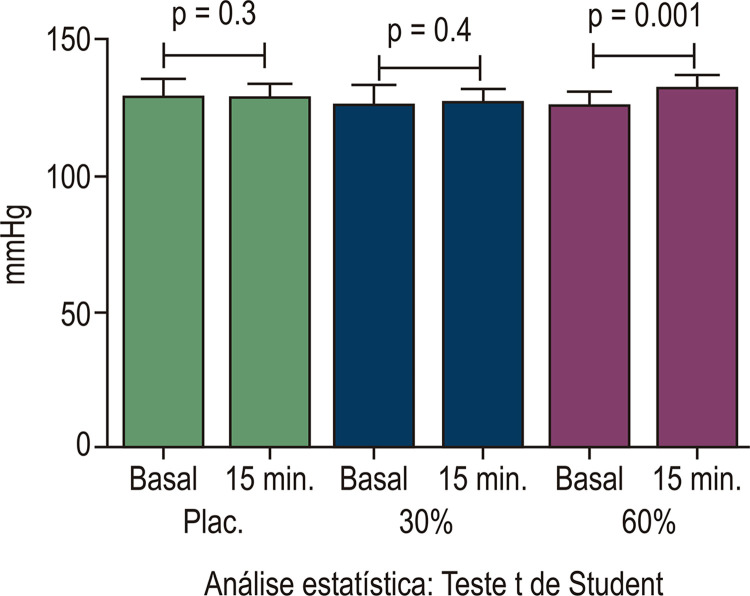
– *Comportamento da PAS no EI com as diferentes cargas. (Basal 124,1 ± 27,4 vs 15 min. 130,6 ± 25,9 mmHg, p=0,001). PAS: pressão arterial sistólica; EI: exercício inspiratório; mmHg: milímetros de mercúrio; min.:minutos. (Fonte: O autor).*

**Figura 6:**
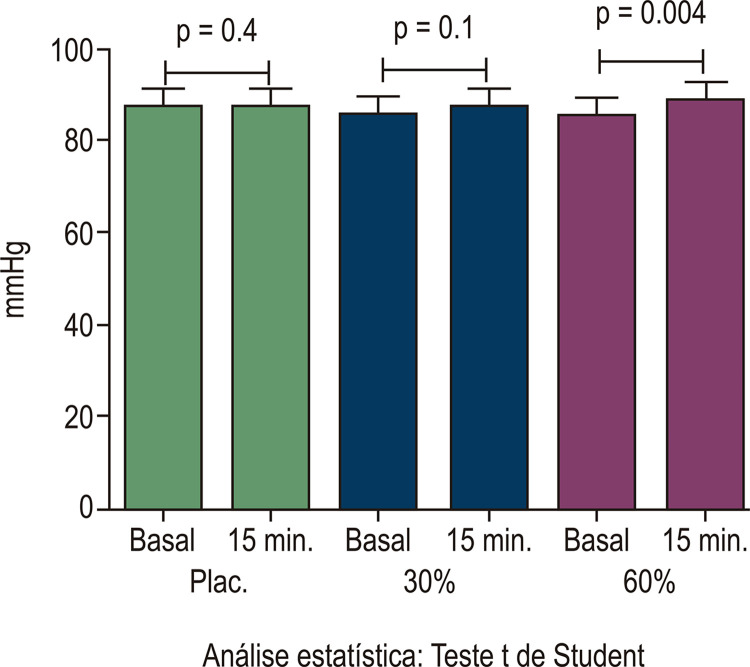
– *Comportamento da PAM no EI com as diferentes cargas. (Basal 85,7 ± 17,9 vs 15 min. 89,2 ± 17,3 mmHg, p=0,004). PAM: pressão arterial média; EI: exercício inspiratório; mmHg: milímetros de mercúrio; min.: minutos. (Fonte: O autor).*

**Figura 7:**
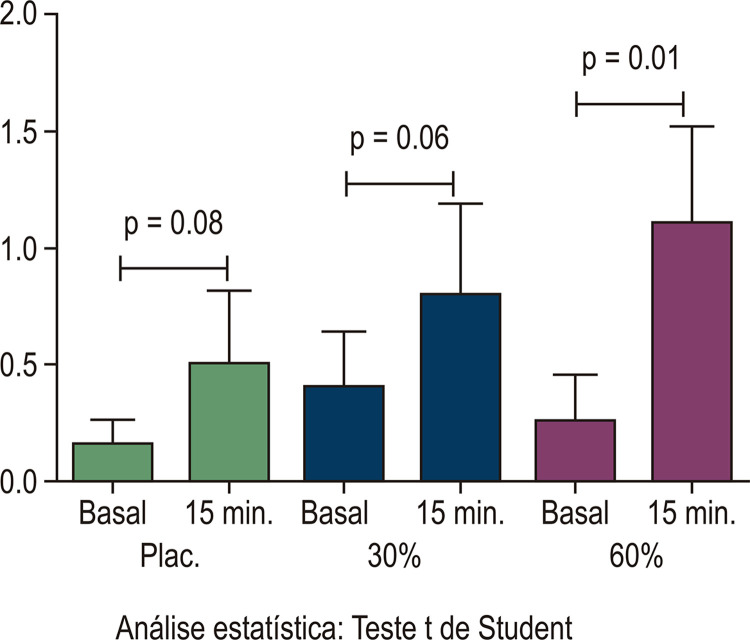
**–** Comportamento do Borg no EI com as diferentes cargas. (Basal 0,3 ± 0,9 vs 15 min. 1,1 ± 1,9, p=0,01). EI: exercício inspiratório; min.: minutos. (Fonte: O autor)

**Figura 8:**
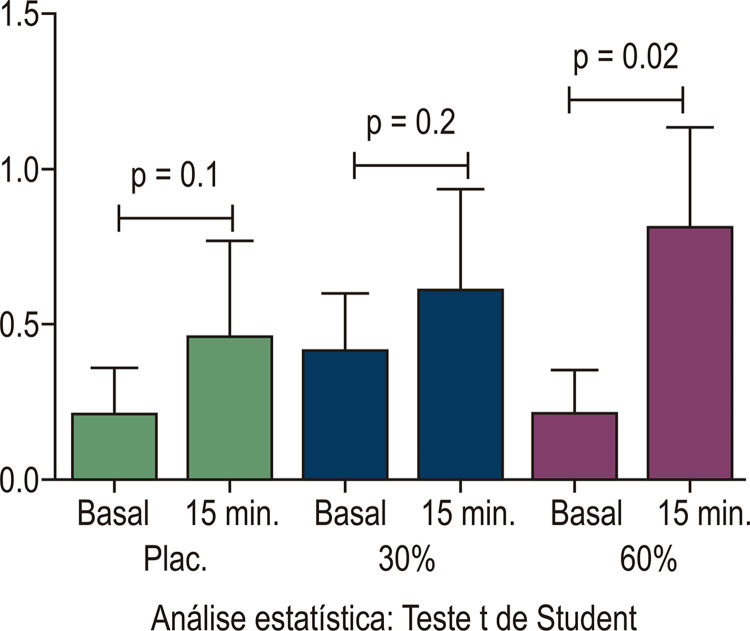
**–** Comportamento da escala subjetiva de dispneia no EI com as diferentes cargas. (Basal 0,2 ± 0,7 vs 15 min. 0,8 ± 1,5, p=0,02). EI: exercício inspiratório; min.: minutos. (Fonte: O autor)

Quando realizado o EI com a C60, houve aumento da pressão arterial sistólica (PAS) (124,1±27,4 vs 130,6±25,9 mmHg; p=0,001) ( Figura 5 ), da pressão arterial média (PAM) (85,7±17,9 vs 89,2±17,3 mmHg, p=0,004) ( Figura 6 ), bem como aumento da escala de esforço percebido de Borg (0,3±0,9 vs 1,1±1,9, p=0,01) ( Figura 7 ) e da escala subjetiva de dispneia (0,2±0,7 vs 0,8±1,5, p=0,02) ( Figura 8 ).

### Correlação

**Figura 9:**
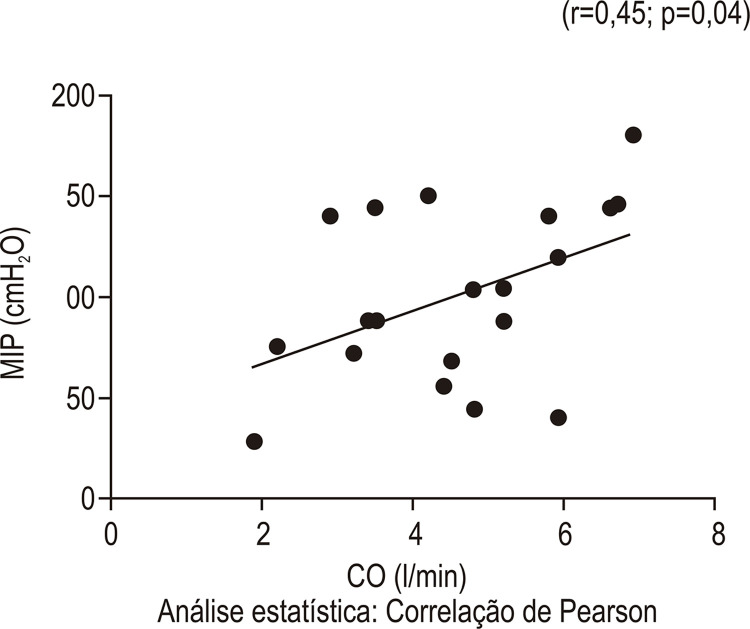
– *Correlação entre o DC basal e a força muscular inspiratória, r=0,45; p=0,04. DC: débito cardíaco; l/min: litros por minuto; PIMáx: pressão inspiratória máxima; cm/H_2_O: centímetros de água.*

Houve moderada correlação entre o DC basal e a força muscular inspiratória (r=0,45; p=0,04) ( Figura 9 ).

## Discussão

O presente estudo é pioneiro ao descrever as alterações na RHC com diferentes cargas de EI em pacientes ambulatoriais com ICFER, utilizando um método não invasivo de monitorização hemodinâmica. Diferentes estratégias de TMI são utilizadas na prática clínica, porém não está claro qual intensidade de treinamento é a mais eficiente.

Houve um comportamento hemodinâmico diferente quando comparadas as cargas placebo, de 30% e 60%. Apenas a FC apresentou resposta semelhante ao EI com as cargas de 30% e 60%, com aumento dessa variável. Já o VS sofreu queda significativa somente quando utilizada a carga de 30%, e o DC aumentou apenas no EI com a maior carga, de 60%. O grupo controle (placebo) não apresentou nenhuma mudança significativa.

Neste estudo foi testada a hipótese de que diferentes cargas de EI poderiam produzir uma resposta hemodinâmica central diferente entre elas. Apesar de já ter sido testada tanto a variabilidade da FC^10^ como os efeitos da fadiga muscular respiratória^11^ através do TMI, este estudo, até então, é o único a verificar a resposta hemodinâmica central de diferentes cargas de EI em pacientes com ICFER.

Para avaliar a resposta hemodinâmica, foi utilizado um aparelho de bioimpedância cardiotorácica, um método de avaliação hemodinâmica não invasiva que, quando comparado a métodos de termodiluição, apresentou elevada correlação.^12^ Mesmo quando utilizada na avaliação de pacientes cardiopatas, como demonstrado no estudo de Villacorta et al.,^13^ a BCT demonstrou acurácia no cálculo do DC, índice cardíaco e VS quando comparada à ressonância magnética cardíaca. Portanto, no presente estudo, foi empregado um método de avaliação hemodinâmica capaz de registrar fidedignamente as mudanças ocorridas ao longo do EI.

A fraqueza muscular inspiratória, presente em cerca de 30 a 50% dos pacientes com ICFER, tem sido reconhecida como um fator que contribui para a limitação ao exercício nesses pacientes, além de possuir valor prognóstico independente.^3 - 5^

Um dos principais estudos com TMI na IC foi realizado por Dall’ago et al.,^9^ onde 32 pacientes foram randomizados em dois grupos (TMI-placebo e TMI 30%). Após 12 semanas de treinamento (7 vezes na semana, durante 30 minutos), os pacientes do grupo intervenção apresentaram aumento significativo de 115% na PImáx, 17% de aumento da captação de oxigênio de pico e 19% de aumento na distância percorrida em seis minutos, além de melhora na qualidade de vida.

Os estudos com treinamento muscular inspiratório, realizados desde 1995 na IC, concentram-se em demonstrar a melhora na força e resistência muscular, melhora da capacidade funcional e na qualidade de vida.^6 , 7^ Porém, as repercussões hemodinâmicas do EI continuam pouco esclarecidas.

### Variáveis hemodinâmicas

A tendência da resposta hemodinâmica, quando um indivíduo saudável é submetido a uma carga resistiva ao exercício, é de elevar a PAS, ao mesmo tempo em que o DC aumentará e, independentemente, os componentes da fórmula desta variável. Em relação à intensidade do exercício, há evidências de que quanto maior a intensidade para o mesmo número de repetições, maior o aumento da FC e da pressão arterial.^14^ De fato, isso ocorreu no presente estudo, pois, ao longo dos 15 minutos de EI, a maior intensidade foi responsável pelos aumentos mais significativos de FC e PAS.

Ainda, o DC para as diferentes cargas resistivas aumentou 15% com a carga de 60% e diminuiu 3% com a carga de 30%.

Sabe-se que o aumento no DC pode ocorrer devido ao aumento apenas da FC, apenas do VS, ou de ambos. No presente estudo, o aumento do DC no EI com carga de 60% ocorreu principalmente devido ao aumento significante da FC, com uma pequena participação do VS, visto que essa variável também aumentou, porém em menor escala, de forma não significativa. Por outro lado, quando utilizada a carga de 30%, o DC apresentou comportamento inverso, com uma pequena diminuição, mesmo havendo aumento na FC. Neste caso, o que parece ter sido determinante para o não aumento do débito foi a queda de 12,5% do VS.

Alguns pesquisadores relatam que, durante o exercício nos pacientes com IC, ocorre um pequeno aumento do VS. Outros demonstram que não ocorre nenhum aumento dessa variável.^14^ No presente estudo, com carga de 60%, a resposta foi um pequeno aumento de 4,5% e uma diminuição no EI com carga de 30%.

A diminuição do VS e o aumento da FC com carga de 30% reportados nesse estudo são semelhantes à repercussão hemodinâmica da manobra de Muller, que também faz com que haja pressão intratorácica negativa. Orban et al.,^15^ estudaram o efeito hemodinâmico da manobra da Muller, sustentada por 12 segundos, em 20 adultos jovens saudáveis e, dentre outros resultados, encontraram diminuição do VS e aumento da FC. Hall et al.,^16^ avaliaram o efeito da manobra de Muller, sustentada por 15 segundos, em 8 pacientes com insuficiência cardíaca congestiva e concluíram que, durante a manobra, ocorre aumento da pós-carga ventricular esquerda e diminuição do volume sistólico, porém a FC não sofreu alterações significativas.

Entretanto, a pressão necessária para a realização da manobra de Muller é em torno de -40 mmHg (-54 cm/H_2_O), e a carga média utilizada durante as sessões de EI foram de -31 cm/H_2_O com 30% e -61 cm/H_2_O com 60%. Desse modo, a carga que mais se aproximou do valor para a realização da manobra de Muller não foi a que apresentou comportamento semelhante à manobra, exceto o aumento da FC.

McConnell e Griffiths,^17^ avaliaram a resposta aguda da FC, PA e PAM a diferentes cargas de treinamento muscular inspiratório (50%, 60%, 70%, 80% e 90%) em 8 atletas. Todas as cargas provocaram aumento da FC, porém apenas a carga de 60% provocou um aumento sustentado da PAM, PAS e pressão arterial diastólica (PAD). Como conclusão, os autores sugerem evidência de uma resposta à ativação do metaborreflexo nesta carga.

O resultado encontrado pelos autores citados acima são semelhantes ao do presente estudo, onde ambas as cargas produziram aumento da FC, porém apenas a carga de 60% gerou aumento significativo na PAS e PAM, o que pode ter ocorrido devido à ativação do metaborreflexo inspiratório.

Essa hipótese está de acordo com outros estudos, em que os autores afirmam que a ativação do metaborreflexo inspiratório se manifesta pelo aumento da FC e da PAM.^18 , 19^

A ativação do metaborreflexo pelo trabalho muscular inspiratório é um fator que contribui para a intolerância ao exercício nos pacientes com IC. Durante o aumento o trabalho respiratório, ocorre redistribuição do fluxo sanguíneo dos músculos periféricos para o diafragma, cerca de 14 a 16% de roubo de fluxo do DC, ocasionando exacerbação da fadiga dos músculos periféricos.^20^

Corroborando os achados do presente estudo, Moreno et al.,^11^ avaliaram o efeito da fadiga muscular respiratória na oxigenação e perfusão dos músculos intercostais e do antebraço em pacientes com ICFER. Após realizarem o exercício inspiratório com carga de 60% até a fadiga, os autores relataram diminuição da perfusão e oxigenação tanto no músculo intercostal quanto no antebraço, e sugerem que isso leva a uma redução do reflexo de perfusão muscular da musculatura periférica, levando à ativação do metaborreflexo inspiratório.

No entando, a longo prazo, Chiappa et al.,^21^ demonstraram que 4 semanas de TMI com carga de 60% é capaz de atenuar o metaborreflexo inspiratório em pacientes com IC e fraqueza dessa musculatura. Os autores também relataram aumento significativo na pontuação de Borg, o que não ocorreu com o grupo controle, que realizou o treinamento apenas com 2% da PImáx.

Tal resultado é similar ao de nosso estudo, onde apenas a maior carga aumentou significativamente a pontuação de Borg, além de elevar também a pontuação na escala subjetiva de dispneia; porém, essa última escala não foi avaliada no estudo de Chiappa et al.^21^

Altas cargas de TMI (60-70%) são recomendadas para promover um melhor efeito em pacientes com IC, e cargas menores (20-40%) são indicadas para pacientes com maior classe funcional.^3^ O presente estudo demonstrou um maior grau de fadiga e dispneia, bem como maiores efeitos sobre a RHC, durante uma sessão única de exercício inspiratório com carga de 60%, o que corrobora essa recomendação e salienta um potencial de risco para indivíduos isquêmicos e com recente descompensação da IC.

Crisafulli et al.,^22^ foram os primeiros a avaliarem a resposta hemodinâmica aguda à ativação do metaborreflexo em seres humanos com IC e a compararem à resposta de indivíduos saudáveis. Para isso, foram selecionados nove pacientes com ICFER e nove voluntários saudáveis. Todos foram submetidos à isquemia pós-exercício. A resposta hemodinâmica, assim como no presente estudo, foi avaliada por impedância cardiográfica. Como resultados, os autores relataram que o aumento na PAS foi semelhante em ambos os grupos, porém o grupo controle obteve aumento da PAS devido ao aumento do DC; já no grupo de pacientes com IC, esse aumento ocorreu devido ao aumento da RVS. Houve ainda aumento do VS no grupo de indivíduos saudáveis e diminuição dessa variável nos pacientes com IC. Os autores sugerem que o aumento da RVS ocorre pela incapacidade dos pacientes com IC de melhorarem o desempenho cardíaco e o VS.

### Correlação entre DC e PImáx

Foi encontrada, em nosso estudo, uma moderada correlação entre DC e PImáx.

Resultado semelhante foi encontrado por Nishimura et al.,^23^ ao avaliarem 23 pacientes com IC. Porém, a correlação encontrada foi entre o índice cardíaco e a PImáx. Naquela época, os autores já sugeriram que a musculatura inspiratória poderia ser dependente da função cardíaca.

Mais recentemente, Filusch et al.,^24^ avaliaram 532 pacientes com insuficiência cardíaca congestiva por meio de cateterismo cardíaco direito e também encontraram moderada correlação entre DC e PImáx. Os autores afirmam que a PImáx, por ser facilmente medida na prática clínica, pode se tornar um parâmetro adicional na monitorização hemodinâmica não invasiva da gravidade da doença.

Meyer et al.,^4^ foram os primeiros a demonstrarem que a força muscular inspiratória possui valor prognóstico independente. Eles acompanharam durante 23 meses 244 pacientes com ICFER e, ao longo desse período, os 57 pacientes (23%) que morreram apresentaram PImáx ainda mais reduzida que o restante da amostra.

Corroborando com os achados de Meyer, Frankenstein et al.,^5^ em um estudo prospectivo com 686 pacientes, evidenciaram que a PImáx pode ser considerada valor prognóstico mesmo naqueles que fazem uso de β-bloqueadores.

### Limitações do estudo

O tamanho da amostra impossibilitou avaliar apenas o grupo com fraqueza muscular inspiratória e, tendo em vista que o presente estudo foi de efeito agudo, não sabemos se esses efeitos são mantidos ou atenuados. São necessárias futuras investigações que avaliem a RHC relacionada ao TMI de forma crônica.

### Aplicabilidade clínica

Esses dados apontam que a resposta hemodinâmica do EI, em suas diferentes propostas de carga resistiva com o resistor de carga linear, poderia ter um potencial de aplicabilidade no tratamento não medicamentoso em pacientes com IC (NYHA II e III), de forma segura, sem efeitos adversos.

## Conclusões

Quando utilizada a carga de 60%, em uma sessão única de exercício inspiratório, foram observadas alterações na RHC. Particularmente aumentaram a frequência cardíaca, o débito cardíaco, a escala de Borg e a sensação subjetiva de dispneia. Já a carga de 30% promoveu diminuição do volume sistólico. O placebo não promoveu mudanças significativas na RHC no presente estudo e, por fim, observou-se uma correlação moderada entre débito cardíaco e força muscular inspiratória.
