# Supplementary material for: Estudo Controlado das Alterações Hemodinâmicas Centrais de uma Sessão de Exercício Inspiratório com Diferentes Cargas na Insuficiência Cardíaca
Source: Arq Bras Cardiol. 2020 May 12;114(4):656–63. [Article in Portuguese] doi: 10.36660/abc.20180375 (PMC9744347; doi:10.36660/abc.20180375)
Supplement: Translation

# Translation: Controlled Study of Central Hemodynamic Changes in Inspiratory Exercise with Different Loads in Heart Failure

**Authors:** Luana de Decco Marchese, Sergio Chermont, Danielle Warol, Lucia Brandão de Oliveira, Sabrina Bernardez Pereira, Mônica Quintão, Evandro Tinoco Mesquita

**Affiliations:** 1 Brazil Universidade Federal Fluminense, Niterói, RJ – Brazil; 2 Brazil Centro Universitário Serra dos Órgãos – Clínica de Insuficiência Cardíaca (CLIC), Teresópolis, RJ – Brazil; 3 Brazil Hospital Santa Martha, Niterói, RJ – Brazil; 4 Brazil Hospital do Coração, São Paulo, SP – Brazil; 5 Brazil Instituto Nacional do Câncer, Rio de Janeiro, RJ – Brazil

**Keywords:** Heart Failure, Muscle Weaknerss, Breathing Exercises, Hemodynamics, Fatigue Syndrome, Chronic, Quality of Life, Exercise Therapy, Exercise Movement Techniques

## Abstract

**Background:**

Inspiratory muscle weakness contributes to exercise intolerance and decreased quality of life in patients with heart failure. Studies with inspiratory muscle training show improvement in inspiratory muscle strength, functional capacity and quality of life. However, little is known about the central hemodynamic response (CHR) during inspiratory exercise (IE).

**Objective:**

To evaluate CHR in a single IE session with different loads (placebo, 30% and 60%) in heart failure.

**Methods:**

Randomized placebo-controlled clinical trial in patients with heart failure with reduced ejection fraction, functional class II and III. Twenty patients aged 65 ± 11 years completed a single session of inspiratory exercise, in 3 cycles of 15 minutes, with a 1-hour washout, involving loads of 30% (C30), 60% (C60) and placebo, using a linear load resistor (PowerBreathe Light). The noninvasive hemodynamic study was performed by cardiothoracic bioimpedance (Niccomo™ CardioScreen®). Statistical analysis was performed with Student’s t-test and Pearson’s correlation, and P≤0.05 was considered significant.

**Results:**

An increase in heart rate (HR) was observed with C30 (64 ± 15 vs 69 ± 15 bpm; p = 0.005) and C60 (67 ± 14 vs 73 ± 14 bpm, p = 0.002). A decrease was observed in systolic volume (SV) with C30 (73 ± 26 vs 64 ± 20 ml; p = 0.004). Cardiac output (CO), on its turn, increased only with C60 (4.6 ± 1.5 vs 5.3 ± 1.7 l/min; p = -0.001).

**Conclusion:**

When using the 60% load, in a single IE session, changes in CHR were observed. HR and CD increased, as did the Borg scales and subjective sensation of dyspnea. The 30% load reduced the SV. (Arq Bras Cardiol. 2020; 114(4):656-663)

## Introduction

Most patients with heart failure (HF) have exercise intolerance, mainly due to symptoms such as dyspnea and fatigue. This low tolerance to physical efforts generates a cycle of physical inactivity and a consequent decrease in quality of life.^1^

In addition to other mechanisms previously described such as excessive ventilatory need, exacerbated muscle ergoreflex and increased sympathetic activity, inspiratory muscle weakness, present in approximately 30 to 50% of patients with heart failure with reduced ejection fraction (HFREF), has been identified as a factor that can contribute to exercise intolerance^2 , 3^ and has an independent prognostic value.^4^

Previous studies have shown that inspiratory muscle training (IMT) results in significant improvements in inspiratory muscle strength, functional capacity, dyspnea and ventilatory response during exercise, besides contributing to the improvement in quality of life of patients with HF.^6 , 7^ However, the ideal training intensity to optimize these results is still unclear. A recent systematic review with meta-analysis suggested that high-intensity IMT is superior to lower loads and does not appear to have any adverse effects.^8^

Most studies have focused on demonstrating the systemic benefits of IMT, but little is known about the central hemodynamic response (CHR) of these patients during inspiratory exercise (IE).^9^ The hypothesis of the present study is that, with higher loads, greater hemodynamic repercussions would be observed. Therefore, this study aimed to assess CHR in a single session of inspiratory exercise with different loads (placebo, 30 and 60%) in HFREF.

### Methods

Randomized, placebo-controlled clinical trial. The load was placed on the linear load resistor, in a way that participants could not see at which level the marker was positioned and were also not informed about the load used.

### Inclusion and exclusion criteria

To meet the objective of this study, 29 patients with HFREF from the Heart Failure Clinic (CLIC) of *Centro Universitário Serra dos* *Órgãos* (UNIFESO) were selected. They all met the following inclusion criteria: clinical diagnosis of heart failure, age over 21 years, Doppler echocardiogram with left ventricular ejection fraction (LVEF) <45% (Simpson method), class II and III by the New York Heart Association (NYHA), stable disease for at least three months, never having undergone or not being treated with IMT. None of the following exclusion criteria were present: clinical (medical) diagnosis of chronic obstructive pulmonary disease, unstable angina, major cardiac arrhythmias, acute myocardial infarction in the last three months, inability to perform the IE session. And yet none of the exclusion criteria for cardiothoracic bioimpedance: massive pleural effusion, anasarca, moderate or severe aortic insufficiency, use of intra-aortic balloon, mean arterial pressure >130mmHg, height <1.20m or >2.30m, weight <30kg or >155kg, and use of pacemakers with sensors to adjust heart rate according to respiratory rate.

### Assessment methods

Collection instruments used were: an analogue manovacuometer (Critical Med®, Brazil), a linear load resistor (PowerBreathe Light®, United States), and a cardiothoracic bioimpedance (CTB) device (Niccomo™ CardioScreen®, Germany).

The inspiratory muscle exercise (IME) sessions were performed according to the randomization made by the Randomizer website, using the linear load resistor for 15 minutes with the following loads: 0 (placebo), 30% and 60% of the maximum inspiratory pressure (MIP) value measured previously by manovacuometry, with a 1-hour washout. To monitor the hemodynamic repercussions, the CTB device was used.

### Inspiratory exercise

As this was the first time participants used the linear load resistor, after the initial evaluation they were instructed on how to perform the IE and then remained at rest for 15 minutes before beginning hemodynamic monitoring.

Following the load randomization done previously (placebo, 30% or 60%), the IE was performed for 15 minutes, with the patient in supine position on a reclining chair, at 45º of elevation. All participants used the same linear load resistor, but an individual filter from the same manufacturer was used and discarded after the experiment.

Throughout the IE, the patient was instructed to perform inspiration and expiration according to the sound signal emitted by a software (Paced Breathing), so all participants performed 15 breaths per minute.^8^ The sessions with the other loads were carried out after a one-hour interval between each. For the IE with placebo, the device's spring was removed, only the unidirectional valve was left, therefore no resistance was present to the patient's inspiration.

### Statistical analysis

The appropriate number of participants to be studied was calculated based on previous publications that showed which intervention, such as the effects of exercise, caused significant changes, such as increased heart rate, among others. For this magnitude of effects and to set statistical power at 0.8 and alpha error at 0.05, the sample should be comprised of 20 individuals.

All data were subjected to Kolmogorov-Smirnov analysis to determine whether or not there was a normal distribution of the sample and data. Hemodynamic variables during IE, in Placebo, 30% or 60% groups, were compared using the Student's t test for paired variables. For the association of independent variables, Pearson's correlation was used. When p values were significant, paired comparisons were made using the Bonferroni test (post-hoc).

The data were transferred to a systematic spreadsheet in Prism GraphPad 5.0 software (GraphPad Software, San Diego, CA). Categorical variables were expressed as absolute numbers. All results were expressed as mean±standard deviation and p values <0.05 were considered statistically significant.

### Ethical considerations

All participants in this study received detailed information about the purpose of the research and the procedures to be performed. The protocol was sent to UNIFESO's Research Ethics Committee and approved under opinion number 420.737, registered at *Plataforma Brasil* .

Before taking part in the study, all participants signed the informed consent form, according to resolution 466/2012 of the National Health Council.

## Results

Among the 29 participants selected for the study, 20 completed the experiment (9 patients refused to participate) ( Figure 1 ). Table 1 describes the demographic, clinical and pharmacological treatment characteristics of the sample.

**Figure 1:** – *CONSORT flowchart.*

**Table 1:** – Sample characteristics

	n=20
Gender	13M\7F
Age (years)	65 ± 11
Weight (kg)	72 ± 14
Height (cm)	164 ± 11
BMI (kg/m2)	26.7 ± 4.4
Ethnicity	Caucasian (7). Brown (6). Afrodescendants (7)
NYHA	Class II (14). Class III (6)
LVEF (%)	37.2 ± 6.3
MIP (cm\H2O)	- 101 ± - 43
MEP (cm\H2O)	95 ± 42
30% load IMT (cm\H2O)	31 ± 11
60% load IMT (cm\H2O)	61 ± 25
**Pharmacological therapy**	
ACE inhibitor, %	55
Diuretics, %	75
β-blocker, %	80

*M: male; F: female; kg: kilogram; cm: centimeters; BMI: body mass index; kg/m^2^: kilograms per square meter; LVEF: left ventricular ejection fraction; MIP: maximum inspiratory pressure; MEP: maximal expiratory pressure; IMT: inspiratory muscle training; cm/H_2_O: centimeters of water; ACE: angiotensin-converting enzyme.*

### Responses of central hemodynamic variables to IE

The central hemodynamic response had a different behavior according to different IE loads in our sample. HR increased with loads of 30% (C30) (64 ± 15 vs 69 ± 15 bpm; p=0.005) and 60% (C60) (67 ± 14 vs 73 ± 14 bpm, p=0.002), but did not change in the placebo mode (P) ( Figure 2 ). There was a decrease in SV when the IE was performed with C30 (73 ± 26 vs 64 ± 20 ml; p=0.004) and there were no changes with placebo and C60 ( Figure 3 ). The DO increased when the IE was performed on C60 (4.6 ± 1.5 vs 5.3 ± 1.7 l/min; p=-0.001) and did not change with placebo and C30 ( Figure 4 ).

**Figure 2:** **–** HR behavior before and at 15 min. of IE with the different loads. HR: heart rate; IE: inspiratory exercise; bpm: beats per minute; plac: placebo; min: minutes. (Source: The author)

**Figure 3:** **–** Behavior of the SV before and at 15 min. of IE with the different loads. SV: systolic volume; IE: inspiratory exercise; ml: milliliter; plac: placebo; min: minutes. (Source: The author)

**Figure 4:** – *Behavior of CO before and at 15 min. of IE with the different loads. CO: cardiac output; IE: inspiratory exercise; ml: milliliter; plac: placebo; min: minutes. (Source: The author)*

### Responses of other hemodynamic variables to IE

In addition to CHR, other hemodynamic variables also changed along the IE on the load C60. Placebo and C30 did not cause any changes in the variables presented.

When the IE was performed on C60, there was an increase in systolic blood pressure (SBP) (124.1 ± 27.4 vs 130.6 ± 25.9 mmHg; p=0.001) ( Figure 5 ), mean arterial pressure (MAP) (85.7 ± 17.9 vs 89.2 ± 17.3 mmHg, p=0.004) ( Figure 6 ), as well as an increase in Borg scale of perceived exertion (0.3±0.9 vs 1.1±1.9, p=0.01) ( Figure 7 ) and the subjective dyspnea scale (0.2±0.7 vs 0.8±1.5, p=0.02) ( Figure 8 ).

**Figure 5:** – *SBP behavior in the IE with the different loads. (Baseline 124.1 ± 27.4 vs 15 min. 130.6 ± 25.9 mmHg, p = 0.001). SBP: systolic blood pressure; IE: inspiratory exercise; mmHg: millimeters of mercury; min.: minutes. (Source: The author).*

**Figure 6:** – *MAP behavior in the IE with the different loads. (Baseline 85.7 ± 17.9 vs 15 min. 89.2 ± 17.3 mmHg, p = 0.004). MAP: mean arterial pressure; IE: inspiratory exercise; mmHg: millimeters of mercury; min.: minutes. (Source: The author).*

**Figure 7:** **–** Borg results in the IE with different loads. (Basal 0.3 ± 0.9 vs 15 min. 1.1 ± 1.9, p = 0.01). IE: inspiratory exercise; min.: minutes.(Source: The author)

**Figure 8:** – *Behavior of the subjective scale of dyspnea in IE with different loads. (Baseline 0.2 ± 0.7 vs 15 min. 0.8 ± 1.5, p = 0.02). IE: inspiratory exercise; min.: minutes. (Source: The author)*

### Correlation

There was a moderate correlation between baseline CO and inspiratory muscle strength (r=0.45; p=0.04) ( Figure 9 ).

**Figure 9:** **–** Correlation between baseline CO and inspiratory muscle strength, r = 0.45; p = 0.04. CO: cardiac output; l/min: liters per minute; MIP: maximum inspiratory pressure; cm/H_2_O: centimeters of water.

## Discussion

This is a pioneer study in describing changes in CHR with different IE loads in outpatients with HFREF, using a non-invasive method of hemodynamic monitoring. Different IMT strategies are used in clinical practice, but it is not clear which training intensity is the most efficient.

There were different hemodynamic behaviors when comparing placebo, 30% and 60% loads. Only the HR showed a similar response to IE with both loads of 30% and 60%, being increases. SV, on the other hand, had a significant drop only when the 30% load was used, and the CO increased only with the highest load, 60%. The control group (placebo) showed no significant change.

In this study, the hypothesis that different IE loads could produce different central hemodynamic responses was tested. Although both HR^10^ variability and the effects of respiratory muscle fatigue^11^ after IMT have already been tested, this study, until then, is the only one to verify the central hemodynamic response of different IE loads in patients with HFREF.

To assess hemodynamic response, a cardiothoracic bioimpedance device was used, which is a noninvasive hemodynamic assessment method that, when compared to thermodilution methods, showed high correlation.^12^ Even when used to assess cardiac patients, as shown in the study by Villacorta et al.,^13^ CTB showed accuracy in the calculation of CO, cardiac index and SV when compared to cardiac magnetic resonance. Therefore, in our study, a method of hemodynamic evaluation capable of reliably recording the changes occurred during IE was used.

Inspiratory muscle weakness, present in about 30 to 50% of patients with HFREF, has been acknowledged as a factor that contributes to exercise limitation, in addition to having an independent prognostic value.^3 - 5^

One of the main studies with IMT in HF was carried out by Dall'ago et al.,^9^ in which 32 patients were randomized into two groups (IMT-placebo and IMT 30%). After 12 weeks of sessions (7 times a week, for 30 minutes), the patients in the intervention group showed a significant increase of 115% in MIP, 17% increase in peak oxygen uptake, and 19% increase in the distance covered in six minutes, in addition to an improvement in quality of life.

Studies with inspiratory muscle training, performed since 1995 in HF, have focused on demonstrating the improvement in muscle strength and endurance, improvement in functional capacity and quality of life.^6 , 7^ However, the hemodynamic repercussions of the IE remain unclear.

### Hemodynamic variables

When a healthy individual is subjected to a resistive load to exercise, the tendency of the hemodynamic response is to increase SBP, at the same time that the CO will increase and, independently, the components of the formula of this variable. Regarding the intensity of the exercise, there is evidence that the greater the intensity for the same number of repetitions, the greater the increase in HR and blood pressure.^14^ In fact, this occurred in the present study because, over the 15 minutes of IE, the highest intensity was responsible for the most significant increases in HR and SBP.

Furthermore, the CO for the different resistive loads increased by 15% with the 60% load and decreased by 3% with the 30% load.

It is known that the increase in CO can occur due to an increase in HR alone, only in SV, or both. In our study, the increase in CO in IE with a 60% load occurred mainly due to the significant increase in HR, with a small participation of SV, since this variable also increased, but on a smaller, non-significant scale. On the other hand, when the 30% load is used, the CO had an inverse behavior and presented a small decrease, even with an increase in HR. In this case, what seems to have been decisive for the non-increase in output was the 12.5% drop in SV.

Some researchers report that, during exercise in patients with HF, a small increase in SV occurs. Others demonstrate that there is no increase in this variable.^14^ In this study, the response to a load of 60% was a small increase of 4.5% and a decrease in IE with a 30% load.

The decrease in SV and the increase in HR with a 30% load reported in this study are similar to the hemodynamic repercussions of the Muller maneuver, which also causes negative intrathoracic pressure. Orban et al.^15^ studied the hemodynamic effects of the Muller maneuver sustained for 12 seconds in 20 healthy young adults and, among other results, found a decrease in SV and an increase in HR. Hall et al.^16^ evaluated the effect of the Muller maneuver sustained for 15 seconds in 8 patients with congestive heart failure and concluded that, during the maneuver, there is an increase in left ventricular afterload and a decrease in systolic volume, but HR did not show significant changes.

However, the pressure required to perform the Muller maneuver is around -40 mmHg (-54 cm/H_2_O), and the average load used during IE sessions was -31 cm/H_2_O with 30% load and -61 cm/H_2_O with 60% load. Thus, the load that came closest to the value for performing the Muller maneuver was not the one with a behavior similar to the maneuver, except for the increase in HR.

McConnell and Griffiths^17^ assessed the acute response of HR, BP and MAP to different loads of inspiratory muscle training (50%, 60%, 70%, 80%, and 90%) in 8 athletes. All loads caused an increase in HR, but only a 60% load caused a sustained increase in MAP, SBP and diastolic blood pressure (DBP). In conclusion, the authors suggest an evidence of a response to the activation of the metaborreflex in this load.

The results found by the authors cited above are similar to the findings in this study, where both loads increased HR, but only the load of 60% caused a significant increase in SBP and MAP, which may have occurred due to the activation of the inspiratory metaborreflex.

This hypothesis is in agreement with other studies, in which the authors state that the activation of the inspiratory metaborreflex is manifested by the increase in HR and MAP.^18 , 19^

The activation of the metaborreflex by inspiratory muscle work is a factor that contributes to exercise intolerance in patients with HF. During the increase in the respiratory work, there is a redistribution of blood flow from the peripheral muscles to the diaphragm, about 14 to 16% of flow theft of CO, causing an exacerbation of fatigue in peripheral muscles.^20^

Corroborating the findings of the present study, Moreno et al.^11^ evaluated the effect of respiratory muscle fatigue on oxygenation and perfusion of the intercostal and forearm muscles in patients with HFREF. After inspiratory exercise with a 60% load until fatigue, the authors reported decreased perfusion and oxygenation in both the intercostal muscle and forearm, and suggested that this leads to a reduction in the muscle perfusion reflex of peripheral muscles, activting the inspiratory metaborreflex.

However, in the long run, Chiappa et al.^21^ demonstrated that 4 weeks of IMT with a 60% load is able to attenuate the inspiratory metaborreflex in patients with HF and muscle weakness. The authors also reported a significant increase in the Borg score, which did not occur in the control group, whose exercise was performed with only 2% of MIP.

This result is similar to that of our study, where only the highest load significantly increased Borg's score, in addition to raising the score on the subjective dyspnea scale; however, this last scale was not evaluated in the study by Chiappa et al.^21^

High IMT loads (60-70%) are recommended to promote a better effect in patients with HF, while lower loads (20-40%) are indicated for patients with a higher functional class.^3^ The present study demonstrated a higher degree of fatigue and dyspnea, as well as greater effects on CHR, during a single session of IE with a 60% load, which corroborates this recommendation and highlights a potential risk for individuals with ischemia and with recent decompensation of HF.

Crisafulli et al.^22^ were the first to assess the acute hemodynamic response to the activation of the metaborreflex in humans with HF and to compare it to the response of healthy individuals. For this, nine patients with HFREF and nine healthy volunteers were selected. All were submitted to post-exercise ischemia. The hemodynamic response, as in the present study, was assessed by cardiographic impedance. As a result, the authors reported that the increase in SBP was similar in both groups, but the control group obtained an increase in SBP due to the increase in CO; in the group of patients with HF, this increase occurred due to the increase in systemic vascular resistance (SVR). There was also an increase in SV in the group of healthy individuals and a decrease in this variable in patients with HF. The authors suggest that the increase in SVR occurs due to the inability of patients with HF to improve cardiac performance and SV.

### Correlation between CO and MIP

In our study, a moderate correlation between CO and MIP was found.

A similar result was found by Nishimura et al.^23^ after evaluating 23 patients with HF. However, the correlation found was between cardiac index and MIP. At that time, the authors already suggested that the inspiratory muscles could be dependent on cardiac function.

More recently, Filusch et al.^24^ evaluated 532 patients with congestive heart failure using right heart catheterization and also found a moderate correlation between CO and MIP. The authors state that, as MIP is easily measured in clinical practice, it can become an additional parameter in noninvasive hemodynamic monitoring of disease severity.

Meyer et al.^4^ were the first to demonstrate that inspiratory muscle strength has an independent prognostic value. They followed up 244 patients with HFREF for 23 months, and the 57 patients (23%) who died over that period had MIP even more reduced than the rest of the sample.

Corroborating the findings of Meyer, Frankenstein et al.,^5^ in a prospective study with 686 patients, showed that MIP can be considered a prognostic value even in patients using β-blockers.

### Study limitations

The sample size made it impossible to assess only the group with inspiratory muscle weakness and, given that the present study had an acute effect, we do not know whether these effects are maintained or attenuated. Further investigations are needed to assess chronic IMT-related CHR.

### Clinical applicability

These data indicate that the hemodynamic response of the IE in its different proposals of resistive load with the linear load resistor could have a potential for safe applicability in non-drug treatment of patients with HF (NYHA II and III), without adverse effects.

## Conclusions

When the 60% load was used in a single session of inspiratory exercise, changes in CHR were observed. Particularly increased heart rate, cardiac output, Borg scale and subjective feeling of dyspnea. The 30% load promoted a decrease in systolic volume. Placebo did not promote significant changes in CHR in the present study and, finally, there was a moderate correlation between cardiac output and inspiratory muscle strength.
